# Distribution and Speciation of Heavy Metal(loid)s in Soils under Multiple Preservative-Treated Wooden Trestles

**DOI:** 10.3390/toxics11030249

**Published:** 2023-03-07

**Authors:** Xiu Zeng, Qian Jin, Panpan Wang, Chengmin Huang

**Affiliations:** 1 Department of Environmental Science and Engineering, Sichuan University, Chengdu 610065, China; 2 Jiuzhaigou Administration Bureau, Jiuzhaigou 623402, China

**Keywords:** wooden trestles, wood preservatives, heavy metal(loid)s, soil contamination, toxicity

## Abstract

The widespread use of wood preservatives, such as chromated copper arsenate (CCA), alkaline copper quaternary (ACQ), and copper azole (CA), may cause environmental pollution problems. Comparative studies on the effect of CCA-, ACQ-, and CA-treated wood on soil contamination are rarely reported, and the behavior of soil metal(loid) speciation affected by preservatives has been poorly understood. Soils under the CCA-, ACQ-, and CA-treated boardwalks were collected to investigate metal(loid) distribution and speciation at the Jiuzhaigou World Natural Heritage site. The results showed that the maximum mean concentrations of Cr, As, and Cu were found in soils under the CCA, CCA, and CCA plus CA treatments and reached 133.60, 314.90, and 266.35 mg/kg, respectively. The Cr, As, and Cu contamination in soils within a depth of above 10 cm was high for all types of boardwalks and limited in the horizontal direction, not exceeding 0.5 m. Cr, As, and Cu in soils were mainly present as residual fractions in all profiles and increased with depth. The proportion of non-residual As in soil profiles under CCA- and CCA plus CA-treatment and exchangeable Cu in CA- and CCA plus CA-treatment were significantly higher than those in the profiles under the other preservative treatments. The distribution and migration of Cr, As, and Cu within soils were influenced by the preservative treatment of trestles, in-service time of trestles, soil properties (e.g., organic matter content), geological disasters (e.g., debris flow), and elemental geochemical behavior. With the CCA treatment for trestles successively replaced by ACQ and CA treatments, the types of contaminants were reduced from a complex of Cr, As, and Cu to a single type of Cu, achieving a reduction in total metal content, toxicity, mobility, and biological effectiveness, thus reducing environmental risks.

## 1. Introduction

Wood is one of the most abundant sustainable biomaterials on Earth [1,2]. However, it is susceptible to biological or chemical decay by biotic and abiotic components [3,4,5]. To protect the structural integrity of wood from the harmful effects of fungi, termites, and various other pests [6,7,8], improve the efficiency of wood utilization, and conserve wood resources and increase the functionality of wood products, various wood preservatives have been persistently invented [9]. Preservative-treated wood is widely used in decks, porches, utility poles, railroad sleepers, bridge piers, fence posts, and picnic tables, among other things [10,11,12].

Chromated copper arsenate (CCA) was once the most widely used wood preservative worldwide [13]. CCA-C, consisting of 47.5% CrO_3_, 18.5% CuO, and 34.0% As_2_O_5_ [14], is an ideal wood preservative with excellent preservative efficiency and a low cost. However, since the beginning of this century, CCA has been banned in some countries due to the leaching of Cu, Cr, and As from CCA-treated woods, and the high ecological risks resulting from these metal(loid)s, particularly Cr and As. As a worldwide shift to alternative copper-based preservative-treated wood products [15], alkaline copper quaternary (ACQ) and copper azole (CA), both of which contain copper in the form of copper oxide mixed with organic cobiocides [16], are seeing increasing use. According to the ratio of the primary active ingredient copper oxide and quaternary ammonium salt and the use of different solvents (amine soluble or ammonia soluble), ACQ can be divided into four subtypes, i.e., ACQ-A, ACQ-B, ACQ-C, and ACQ-D [17], and the most widely used in China is ACQ-D. CA contains triazole instead of quaternary ammonium salt used in ACQ. CA is divided into boron-containing formulations (CA-A) and boron-free formulations (CA-B). The current market basically uses boron-free formulations because boron is easily lost [18]. Currently, the type of preserved woods used in China mainly includes CCA-treated wood (accounting for ~85–95%), a small amount of ACQ-treated timber (10%–15%), and a tiny amount of CA-preserved wood [19,20].

The extra entrance of Cr, As, and Cu in CCA-treated wood into the surrounding environment cannot be ignored [21,22,23,24,25]. This not only directly affects the preservative effect, but also the persistence of Cr, As and Cu, producing a risk and threat to environmental security and human health [26,27,28,29]. Although the new environmentally friendly copper-based preservatives exclude Cr and As, the leaching of Cu from the treated wood is still unavoidable [30,31,32]. It has been shown that the leaching rate of Cu from ACQ-treated wood is up to 15 times higher than that in CCA-treated timber [33]. Using X-ray fluorescence spectroscopy, 6.92–19.54% and 9.38–22.46% Cu leaching from ACQ-treated wood and CA-treated wood, respectively, were observed [34]. Despite Cu causing less harm than Cr and As, increased Cu content in the environment may cause damage to aquatic organisms [35,36].

It is well known that Cr, As, and Cu can accumulate and persist for a long time after entering soils and may harm human health through the food chain or airborne dust [37,38,39]. In recent years, numerous studies on the characteristics of Cr, As, and Cu contamination in soils from CCA-treated wood are essential for understanding the soil contamination processes of CCA-treated wood and for contamination management [15,21,24,25,40,41,42,43,44]. However, the contamination processes and behavior of ACQ and CA, so-called “environmentally friendly preservatives”, have yet to be determined. In addition, previous studies on metal(loid) contamination caused by a single type of preserved wood mainly focused on accumulation characteristics and spatial distribution without evaluating soil metal(loid) pollution and potential risk analysis. Meanwhile, with the more recent iterations of preserved wood, studies have not been conducted on whether cumulative effects occur when different wood preservatives are successively introduced into the soil environment.

Jiuzhaigou National Natural Reserve (JNNR) was listed as a World Natural Heritage site by UNESCO in 1992. Over 70 km of wooden plank roads have been built to preserve natural scenery in Jiuzhaigou since 2001. CCA-C, ACQ-D, and CA-B treated wood planks were constructed during 2001–2012, 2013–2017, and 2018–2022, respectively. Therefore, there is an urgent need to evaluate the metal(loid) contamination situation and the ecological risk associated with the presence of a large number of preservative-treated wooden trestles in the Jiuzhaigou scenic area to protect the natural ecosystem.

The As concentrations on the surfaces of in-service CCA-treated wood planks in the JNNR have been investigated using a portable XRF analyzer [45], but lacked attention to the contamination of soils triggered by CCA, ACQ, and CA preservatives. In this study, the content of major contaminants (Cr, As, Cu) and their speciation distribution in soil profiles and soil physicochemical properties (pH, organic matter content) were determined under CCA-, ACQ-, and CA-treated wood planks in the JNNR. The objectives of this study were: (1) to investigate the accumulation and distribution characteristics of soil heavy metal(loid)s in different types of preservative-treated wood at various in-service ages; (2) to determine the metal speciation distribution in the soil profile; and (3) to provide data to support the selection and management of preserved wood types in ecologically sensitive areas where preservative-treated wood panels are heavily used.

## 2. Materials and Methods

### 2.1. General Description of the Study Area

**Figure 1:**
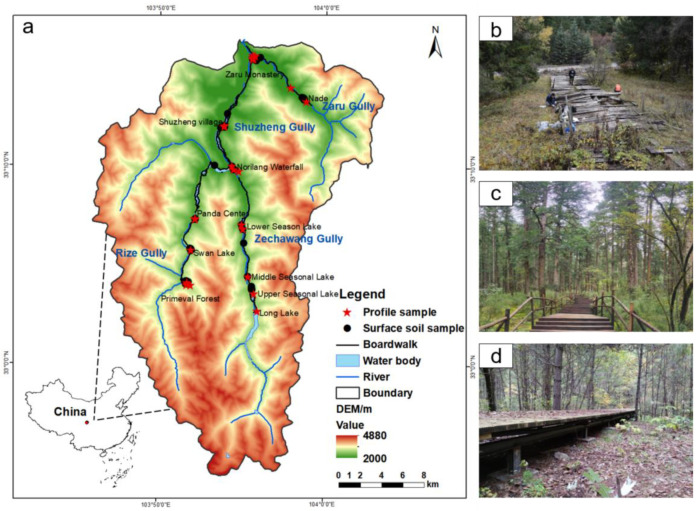
Distribution of boardwalks treated with wood preservatives (**a**) and sampling sites (**b**), CCA treatment, Panda Center; (**c**), ACQ treatment, Primeval Forest; (**d**), CA treatment, Zaru Monastery) in the Jiuzhaigou National Natural Reserve, Southwest China.

Jiuzhaigou National Natural Reserve, situated in northern Sichuan Province, Southwest China, mainly consists of highland travertine lakes, travertine waterfalls, and other typical karst landscapes, covering an area of 643 km^2^ (Figure 1). The altitude of the tourist area is 2000–3100 m above sea level. The scenic route is shaped like a “Y” and is approximately 30 km long. The mean annual temperature and precipitation are 7.3 °C and 622 mm, respectively. Most of the yearly rainfall is concentrated from May to September, ~150 days. The soil texture is mainly sandy loam [46]. Due to various geological disasters, e.g., landslides and debris flows, vegetation and soils in the area were damaged to varying degrees, resulting in rockfall accumulation or exposure of limestone bedrock.

As one of the most attractive scenic spots in China, the JNNR receives millions of domestic and international tourists [47]. To ensure the safety of tourists and reduce the impact of human activities on the ecosystem, more than 70 km of wooden plank roads have been set up in the JNNR since 2001 (Figure 1). For twenty years, three types of preservative-treated planks have been successively replaced. The CCA-treated planks were installed in Jiuzhaigou from 2001 to 2012. Subsequently, ACQ-treated wood was used for building from 2013 to 2017. Since the JNNR was struck by a strong earthquake (Jiuzhaigou earthquake, Ms = 7.0) in 2017, increasing landslides, debris flows, and other natural disasters have resulted in severe damage to infrastructure and public facilities, including the boardwalk in the scenic area. Therefore, as one of the critical projects for restoration and reconstruction, ~73 km-long plank roads with CA treatment were upgraded at the original site from 2018 to 2022.

### 2.2. Sample Collection

Samples were collected at 0–2 cm and 0–10 cm to avoid the dilution effect of contaminant concentrations when collecting 0–10 cm soil. Therefore, a total of 135 topsoil samples (including 0–2 cm soils (N = 42) and 0–10 cm soils (N = 93)) were collected for the different types and service durations of preservative-treated boardwalks (Table 1). Each sample is a combination of five subsamples from the vicinity of 10 m. One hundred and fifteen profile soil samples were collected under the preservative-treated wood at depths of 0–2 cm, 2–5 cm, 5–10 cm, 10–20 cm, 20–30 cm, and 30–40 cm. Some profiles were only excavated to the gravel horizon or bedrock within a depth of 30 cm. To determine the background levels of metal(loid)s in the JNNR, seven surface soil (0–2 cm), eleven surface soil (0–10 cm) and twenty-one soil profile samples at the higher position were collected from sites at least 20 m away from the boardwalks (Table 1). In addition, eight, one, and five CCA-, ACQ-, and CA-treated boards used in JNNR were collected, respectively.

### 2.3. Soil and Wood Sample Analysis

The collected soil samples were air-dried at room temperature. Rock gravel, debris, and plant residues were picked out and weighed separately. A portion of the dried soil sample was weighed, ground, and passed through a 2 mm sieve for pH determination. A portion of the sample was ground until it passed through a 0.15 mm sieve for soil total heavy metal(loid) analysis, heavy metal(loid) speciation analysis, and organic matter content measurement. The boardwalks were sawed into small pieces and then placed in an oven at 60 °C for 48 h. They were ground into powder, passed through a 0.25 mm sieve, and preserved for use.

In the laboratory, the soil samples were digested with a microwave digestion (GT-400, Preekem) method using HCl-HNO_3_-HF-HClO_4_, and the contents of Cr, As, and Cu were determined using inductively coupled plasma mass spectrometry (ICP-MS, NexION 300, PerkinElmer). The wood samples were digested by HNO_3_ and the metal(loid) content was determined by ICP-MS. Soil pH was measured using potentiometry at a 1:2.5 (soil: water) ratio with a pH meter. The soil organic matter (SOM) content was analyzed by the Walkley and Black method through wet oxidation with K_2_Cr_2_O_7_ [48].

Heavy metal(loid)s speciation in profile soils was sequentially extracted with the BCR sequential extraction procedure [49,50]. The four sequential extraction fractions are the exchangeable fraction, reducible fraction, oxidizable fraction, and residual fraction. The heavy metal fractions are shown as a percentage of the total extractable content (%). The determination of the residual fraction was consistent with the total heavy metals. For quality control, the GSS-9 material published by the Institute of Geophysical and Geochemical Exploration, Beijing, China, was used simultaneously during the total and speciation analysis of heavy metals.

### 2.4. Statistical Analysis

ArcGIS 10.7 was used to map the spatial distribution of sampling sites. IBM SPSS Statistics 26 was used for statistical analysis of heavy metal(loid) concentrations and soil properties, one-way ANOVA (*p* < 0.05), and correlation analysis. In addition, the distributions of heavy metal contents, fractions, and soil physicochemical properties in the soil were drawn by Origin 2022.

## 3. Results

### 3.1. Cr, As, and Cu Concentrations in Various Preservative-Treated Wooden Boardwalks

Although the CCA-treated wooden boardwalks have been used for 10–19 years, a large amount of metal(loid)s were still retained in the woods in the JNNR (Table 2). Studies have shown that the preservative retention rate in preservative wood is about 75~95% after 43~1 years of exposure [51,52,53,54]. In this study, the average contents of Cr, As, and Cu in CCA-treated trestles were 2782, 904, and 1561 mg/kg, respectively, indicating that the retention of As was the lowest and Cr was the highest. As is most easily leached from CCA-treated wood and Cr is relatively stable [23,55]. Low Cr and As concentrations were measured in the ACQ-treated trestles relative to the CCA-treated trestles, in contrast with the high Cu content of 5234 mg/kg in Sample W-ACQ-1 from the JNNR. The mean Cu content was 5415 mg/kg in the CA-treated boardwalks (W-CA-1 to W-CA-5) sampled from the JNNR with the lowest Cr and As contents relative to CCA-treated and ACQ-treated woods (Table 2), of which the highest Cu content exceeding 8000 mg/kg was found in fresh Sample W-CA-2.

High concentrations of Cr, Cu, and As were also observed in CCA-treated wood from other parts of the world, with averages of 5944, 6726, and 2742 mg/kg, respectively. Notably, the highest levels were observed in W-CCA-15, a wood pile erected at sea and approximately 25 years old, with Cr, Cu, and As contents of 14,500, 20,700, and 7300 mg/kg, respectively. The Cr, As, and Cu contents from CCA-treated wood are strongly heterogeneous due to various intended usages, realistic usage scenarios, and initial treatment concentrations of the wood. In addition, ACQ- and CA-treated wood from other regions had higher levels of Cu with significant variations in Cu concentration, while As and Cr remained relatively stable with low concentrations. In conclusion, except for the preserved wood used under harsh conditions, the contents of Cr, As, and Cu in different preserved stacks from the JNNR were comparable to those in other regions.

### 3.2. Metal(loid) Concentrations in Surface Soils in the JNNR

#### 3.2.1. Physicochemical Properties and Metal(loid)s in Surface Soils

**Figure 2:**
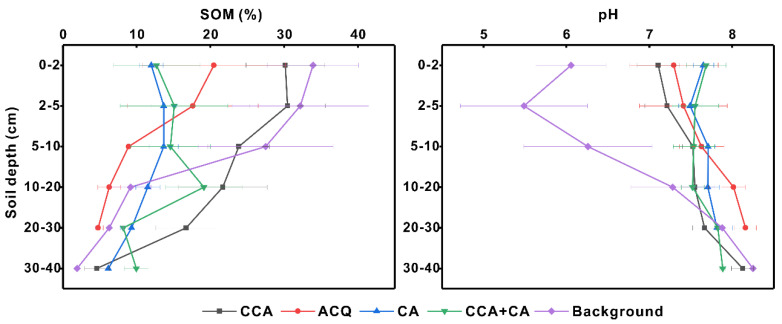
Averaged SOM and pH values within soil profiles under different preservative–treated boardwalks.

In surface soils at a depth within 0–2 cm, the mean values of SOM beneath the CCA, ACQ and CA-treated as well as CCA plus CA-treated trestles were 29.51%, 23.08%, 14.63%, and 18.29%, respectively, while the mean SOM in background samples was 39.74% (Table 3). In surface soils at a depth within 0–10 cm, SOM values under these four treatments were 30.07%, 25.56%, 18.33%, and 18.96%, respectively, while the mean background value was 34.84%. The SOM content at depths of 0–2 cm and 0–10 cm showed the same pattern under different trestles. The background SOM was significantly higher than that of CA- and CCA plus CA-treated soils (*p* < 0.05) and insignificantly different from that of the other preservative-treated soils. This phenomenon may be because the surface humus of soils was removed when constructing the wooden walkways, resulting in a reduction in the SOM, while the accumulation of biomass, such as dead leaves and mosses, increased the OM content gradually with the in-service duration of wood planks. SOM contents decreased with increasing soil depth in all profiles (Figure 2), including the background sample (more details are presented in Table S1). Likewise, the averaged SOM value also increased in surface soils at a depth of 10 cm or shallower within the profile with increasing in-service age of the boardwalks from CA-treatment (0–3 years) to CCA-treatment (8–19 years), and the content in background soils was the highest.

In surface soils at a depth within 0–2 cm, the mean pH values in soils under the CCA-, ACQ-, CA-, and CCA plus CA-treated wooden boards as well as background soils were 7.20, 7.26, 7.48, 7.49, and 6.08, respectively, and were 7.23, 7.13, 7.5, 7.52, and 6.23, respectively in surface soils at a depth within 0–10 cm (Table 3). Most of the samples were neutral to alkaline in pH. The pH of the background was significantly lower than that in the rest of the soils at depths of 0–2 cm and 0–10 cm, and the difference in pH was insignificant between the soils under various preservative treatments. The pH value within the soil profiles generally increased with increasing soil depth (Figure 2). Detailed information was provided in the Supplementary Materials (Table S2). Similarly, the pH value in background soils within a depth of 10 cm was significantly lower (*p* < 0.05) than that in soils under the four wood treatments. The significantly low pH value in the background soils in the JNNR is because (1) a higher organic matter content, derived to a great degree with respect to the alpine elevation of the study area, appeared in the background soils [59,60]. Higher organic matter corresponds to high levels of humic acid, xanthic acid, and low molecular weight organic acids [61,62]; and (2) the use of alkaline materials, such as cement during the construction of trestles, might increase the soil pH [63].

**Table 3:** Metal(loid)s concentrations in surface soils under boardwalks in the JNNR, Southwest China.

Surface Soil Types	Preservative Type	Number	Cr	As	Cu	OM	pH
			mg/kg	mg/kg	mg/kg	g/kg	
SS2(0–2 cm)	CCA	13	141.77Aa ± 61.31	252.75Aab ± 302.00	193.16Aab ± 168.32	29.51Aab ± 11.53	7.20Aa ± 0.59
	ACQ	3	69.79Abc ± 30.44	110.94Aabc ± 69.12	248.53Aab ± 354.20	23.08Aab ± 20.03	7.26Aa ± 0.54
	CA	11	61.05Abc ± 15.74	69.92Abc ± 150.86	241.63Aab ± 146.61	14.63Ab ± 8.01	7.48Aa ± 0.57
	CCA plus CA	8	92.57Aab ± 44.11	324.79Aa ± 378.47	465.44Aa ± 137.07	18.29Ab ± 15.56	7.49Aa ± 0.7
	Background	7	32.34Ac ± 20.85	12.77Ac ± 9.02	14.59Ab ± 4.85	39.74Aa ± 15.70	6.08Ab ± 0.79
SS10(0–10 cm)	CCA	14	133.60Aa ± 64.49	314.90Aa ± 263.64	151.92Aab ± 113.56	30.07Aab ± 11.93	7.23Aa ± 0.61
	ACQ	10	67.43Abc ± 23.30	62.60Aab ± 40.37	187.53Aa ± 205.64	25.56Aab ± 25.32	7.13Aa ± 1.16
	CA	14	67.05Abc ± 14.88	31.67Ab ± 18.22	136.23Aab ± 146.76	18.33Ab ± 8.94	7.52Aa ± 0.38
	CCA plus CA	11	94.97Ab ± 16.88	74.90Ab ± 46.66	266.35Aa ± 213.42	18.96Ab ± 8.51	7.52Aa ± 0.68
	Background	11	49.06Ac ± 22.61	16.34Ab ± 7.71	18.12Ab ± 5.05	34.84Aa ± 18.55	6.23Ab ± 0.77
Risk screening values [64]				20	2000		

For capital letters, mean values followed by different depths under the same preservative treatment differ significantly with *p* < 0.05. For lowercase letters, mean values followed by different preservative treatments at the same depth differ significantly with *p* < 0.05.

The mean Cr concentrations in the surface soil samples within a depth of 2 cm under the CCA, ACQ, CA, and CCA plus CA treatments were 141.77, 69.79, 61.79, and 92.57 mg/kg, respectively, which were higher than the background value (32.34 mg/kg); the Cr content in the surface soils under the CCA and CCA plus CA treatments was significantly higher than the mean value in the background soils (*p* < 0.05) (Table 3). The As concentrations under the CCA, ACQ, CA, and CCA plus CA treatments were 252.75, 110.94, 69.92, and 324.79 mg/kg, respectively; 100%, 100%, 81.82%, and 100% in soil samples higher than the soil risk screening values, of which the CCA- and CCA plus CA-treated values differed significantly from the background values (12.77 mg/kg) (*p* < 0.05). Cu at high levels in the soil under all treatments with 193.16, 248.53, 241.63, and 465.44 mg/kg, and the content of CCA plus CA reached a significant difference level from that in the background soil samples (14.59 mg/kg) (*p* < 0.05).

The background values of Cr, As, and Cu in surface soils at a depth of 0–10 cm were 52.39, 20.93, and 19.85 mg/kg, respectively. The average Cr levels in soil samples under the treatments of CCA, ACQ, CA, and CCA plus CA were 133.60, 67.43, 67.05, and 95.73 mg/kg, respectively, which were higher than those in background soil samples. The mean contents of Cr in CCA-treated and CCA plus CA-treated samples were significantly higher than those in the background soils (*p* < 0.01). The average concentration of As in each type of surface soil sample (314.9, 62.6, 31.6 7, 74.90 mg/kg for CCA, ACQ, CA, and CCA plus CA treatments, respectively) was higher than that in the background soil and risk control standards [64]. Among them, 100%, 100%, 81.82% and 100% of soil samples under the boardwalks treated by CCA, ACQ, CA and CCA plus CA exceeded the risk control standard, respectively. The average As concentration in the CCA-treated samples was the highest among all treatments and significantly higher than that in the CA, CCA plus CA, and background samples (*p* < 0.05). Unlike Cr and As, the highest Cu values were found in the soil samples affected by the CCA plus CA treatment. The average contents of surface soil Cu under the wooden boards treated with CCA, ACQ, CA, and CCA plus CA were 7.7, 9.5, 6.9, and 13.5 times higher than the background values, respectively.

The Cr, As, and Cu contents in soils at a depth between 0 and 2 cm were generally higher than those in soils at depths between 0 and 10 cm under the corresponding treatments (Table 3). A higher concentration in the shallower horizon than in the deeper horizon with the soil profile is often observed when the heavy metal(loid)s in the soils originate mainly from the external environment [65]. This might be related to the high organic matter content of the surface layer. It was found that organic matter content was significantly and positively correlated with As and Cr contents in soils, and organic matter is prone to form complexes with heavy metal ions, thus reducing their ability to migrate downward [66]. This supports the concept that there are low levels of Cu and Cr in soluble or exchangeable form in high organic matter soils and much higher levels in tightly bound organic matter [67]. Furthermore, Cr, As, and Cu contents exhibited the same variation among soils under different boardwalks in both surface soil samples, while the difference between the two types of surface soils with different depths was not significant (Table 3).

#### 3.2.2. Lateral Distribution of Cr, As, and Cu in Surface Soils

**Figure 3:**
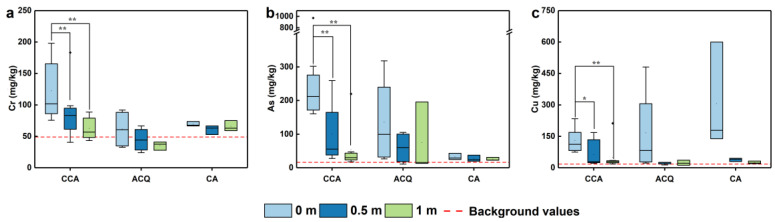
Lateral distribution of Cr (**a**), As (**b**), and Cu (**c**) concentrations in surface soils at a depth of 0–10 cm under CCA-, ACQ-, and CA-treated wooden walkways. *, ** indicate significant (*p* < 0.05) and highly significant (*p* < 0.01) differences.

The distribution of Cr in the soils at a distance within 0 and 1 m near all treatment boardwalks in this study showed a decreasing trend in the horizontal direction (Figure 3a). The pattern of averaged Cr contents in surface soils related to the CCA- and ACQ- boardwalks was similar, with the maximum content occurring at 0 m (beneath the boardwalks), and decreased with increasing distance from the boards. The average Cr content in the surface soils at the horizontal distances of 0, 0.5, and 1 m for the CCA-treated boardwalks was 122.54, 74.28, and 62.56 mg/kg, respectively, and the soil Cr content at a distance of 1 m significantly decreased by 48.95% (*p* < 0.01) relative to that at 0 m. The Cr content in surface soils near the CA-treated boardwalks insignificantly varied with increasing horizontal distance and was close to the background Cr concentration due to the low Cr content in the CA-treated boards.

The As content was highest under the CCA-treated boardwalks and decreased with increasing horizontal distance from 0, 0.5, and 1 m, with average As contents of 211.48, 100.64, and 30.49 mg/kg, respectively, and decreased significantly by 52.41% (*p* < 0.01) and 85.58% (*p* < 0.01) at 0.5 and 1 m compared to 0 m (Figure 3b). The higher As content in the ACQ treatment corresponds to the Cr content, perhaps because the CCA influenced the sampling site. The higher As content in soils under the ACQ treatment corresponds to the Cr content, most likely because the CCA influenced the sampling site. The As concentration in the soils under the CA-treated boardwalks was close to the background value, attributed to the low As contents in CA-treated boardwalks (Table 2).

The mean Cu content in the surface soils under different preservative-treated boardwalks decreased with increasing distance in the horizontal direction (Figure 3c). The averaged soil Cu content under the boards in a descending order is: CA > ACQ > CCA-treatments, in accordance with the order of Cu contents in these preservatives-treated boardwalks (Table 2) and leaching of Cu specifically in different preservatives-treated boardwalks [34]. If the two outliers at the distance from 0.5 and 1 m away from the CCA-treated board were excluded, the average contents of surface soils near CCA-treated boardwalks at a distance of 0, 0.5 and 1 m from the wooden plank were 129.66, 62.58 and 27.36 mg/kg, respectively, and they were significantly decayed by 51.74% (*p* < 0.05) and 78.90% (*p* < 0.01) at 0.5 and 1 m relative to that under the CCA-treated boards, respectively, probably due to the horizontal migration (~ 0.5 m) of Cu after long-term entrance into the soils as Cr and As. The distribution pattern of Cu under the ACQ and CA treatments was similar, in that extremely high Cu contents concentrated in surface soils under the boards, while a sharp reduction appeared at a distance of 0.5 and 1 m away from the boards (Figure 3c).

### 3.3. Vertical Distribution of Metal(loid) Concentrations

**Figure 4:**
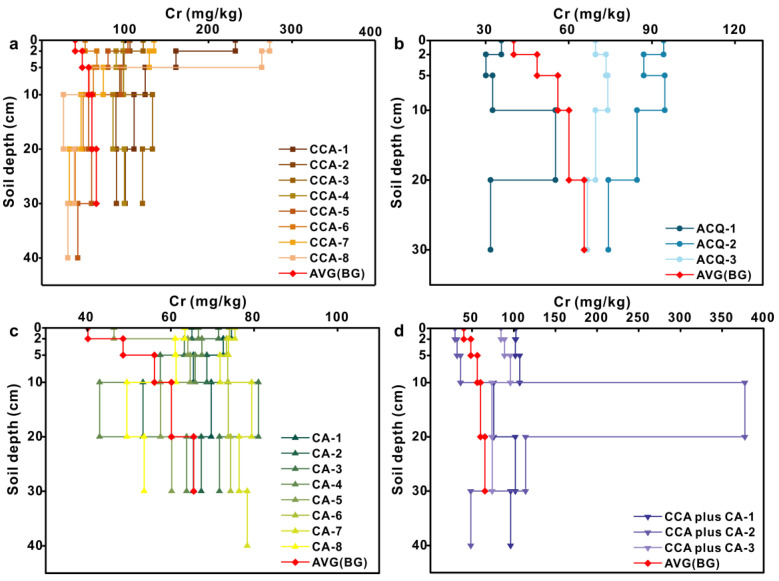
Vertical distribution of Cr concentration in soil samples under wooden walkway with four treatments: (**a**) CCA treatment; (**b**) ACQ treatment; (**c**) CA treatment; (**d**) CCA plus CA treatment.

Compared with the background profile, Cr was mainly enriched in the 0–5 cm soil layer and decreased with increasing soil depth in CCA-treated profiles (Figure 4). Detailed information was provided in the Supplementary Materials (Table S3). The Cr content exhibited significant increases of 249% (*p* < 0.01), 154% (*p* < 0.01), and 56% (*p* < 0.05) at depths within 0–2, 2–5, and 5–10 cm relative to background samples at the same depth, respectively. The mean soil Cr content of each soil layer indicated that Cr might leach out from the CCA-treated woods into soils deeper than 30 cm. The average Cr content of the soils under ACQ- and CA-treated boardwalks gradually decreased with increasing soil depth in contrast with a gradual rise in the background soil profiles (Figures 4b and 4c). For the CCA plus CA treatment, the Cr contents decreased with the increasing soil depth within the profiles except for CCA plus CA-2.

**Figure 5:**
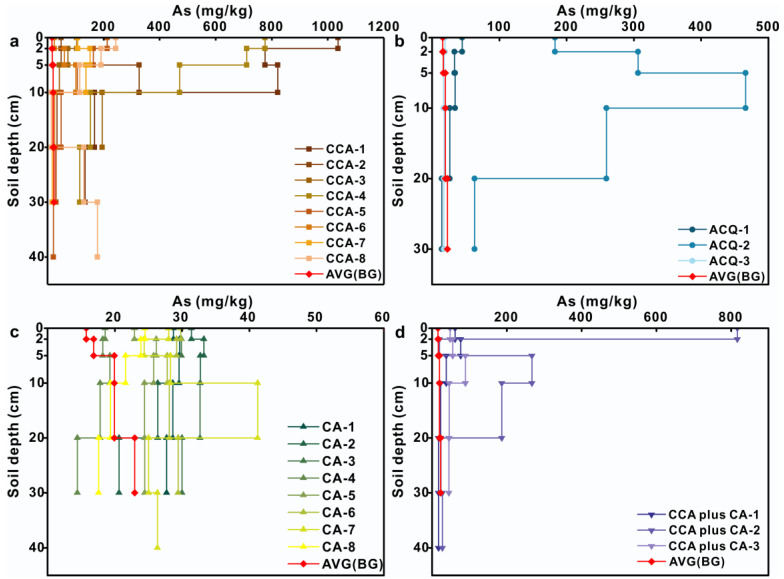
Vertical distribution of As concentration in soil samples under wooden walkway with four treatments: (**a**) CCA treatment; (**b**) ACQ treatment; (**c**) CA treatment; (**d**) CCA plus CA treatment.

It is obvious that the As content was mainly enriched at a depth of 0–10 cm in the soils under CCA-treated boards compared to that in the background profile and gradually decreased to the background value with increasing soil depth (Figure 5). Detailed information was provided in the Supplementary Materials (Table S4). The average As content at depths of 0–2 cm, 2–5 cm, 5–10 cm, 10–20 cm, and 20–30 cm reached 20, 16, 13, 4, and 4 times as high as that in the background content at the corresponding depths, respectively. The mean As contents in the soils under the ACQ- and CA-treated boardwalks were close to the background value and did not change significantly with increasing soil depth, except for the ACQ-2 profile. The As content in the CCA plus CA-treatment profiles decreased with increasing soil depth, except for CCA plus CA-2. Compared with the soil risk screening values, the As contents in the profiles of the ACQ- and CA-treatments were close to each other except for the ACQ-2 profile, which was less hazardous to the environment. The As contents in the CCA-treatment and the CCA plus CA-treatment far exceeded the soil risk screening values, and therefore posed a high environmental risk.

**Figure 6:**
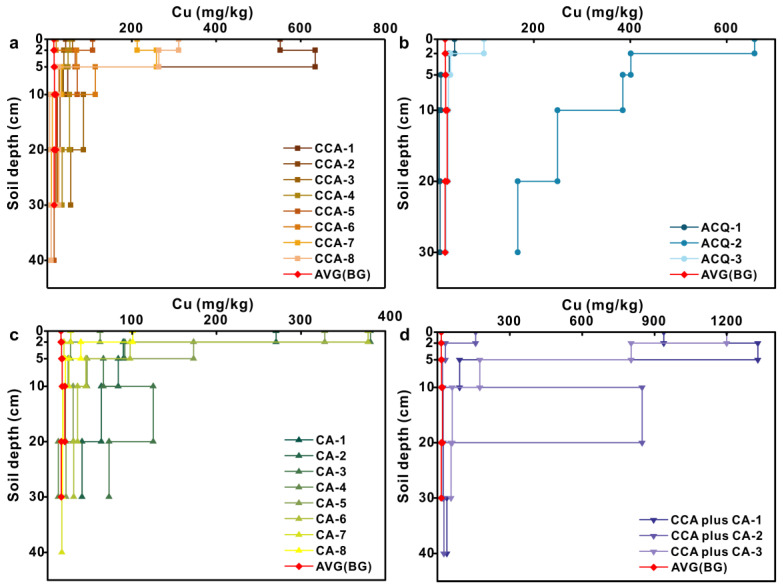
Vertical distribution of Cu concentration in soil samples under wooden walkway with four treatments: (**a**) CCA treatment; (**b**) ACQ treatment; (**c**) CA treatment; (**d**) CCA plus CA treatment.

In the background profiles, Cu concentrations varied slightly with soil depth, ranging from 4.95 to 23.89 mg/kg (Table S5). In general, the Cu concentration within the soil profiles under the four treatment boardwalks decreased with increasing soil depth and dominantly accumulated in the surface soil layer at a depth of 5 cm or shallower (Figure 6). In the soil profiles under the CCA-treated boardwalks, the Cu concentration in the 2–5 cm soil layer was higher than that in the 0–2 cm layer, indicating that Cu might migrate downward owing to prolonged rainwater leaching over 10 years. Among all profiles, the highest Cu concentration occurred within the CCA plus CA-1 profiles. Overall, the Cu concentrations in the profiles under all four treatments did not exceed the soil risk screening values and therefore had a low environmental hazard.

### 3.4. Metal Fractionation and Mobility in Vertical Directions

**Figure 7:**
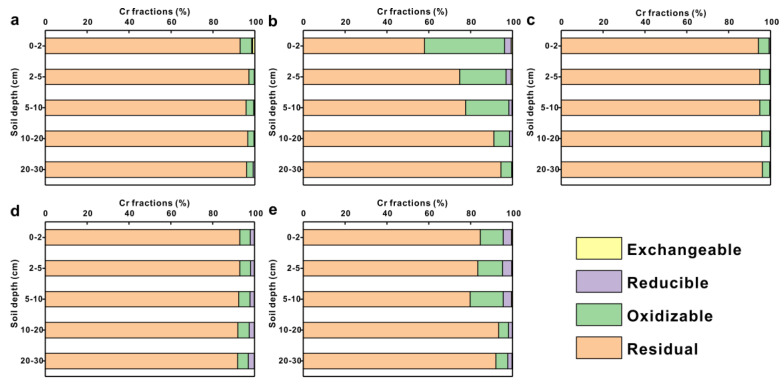
Percentage of exchangeable, reducible, oxidizable, and residual fractions of total Cr in soil profiles under different preservative–treated planks: (**a**) Background profile; (**b**) CCA-5; (**c**) ACQ-3; (**d**) CA-6; (**e**) CCA plus CA-3.

In the background profile, Cr dominated the residual fraction, accounting for 92.99–97.17% of the total content (Figure 7a), and the percentages of each fraction were constant across the profiles. Detailed information about Cr content of each fraction in the soil profiles was presented in the Supplementary Materials (Table S6). Overall, the proportions of each fraction in the soil profiles with four preservative treatments were in the following order of magnitude: residual > oxidizable > reducible > exchangeable, indicating that the residual fraction was still dominant and expressed an increasing trend with increasing profile depth, although the Cr in the residual fraction decreased compared with the background profiles (Figure 7b–e). In addition, the oxidizable fractions in the CCA and CCA plus CA treatment soil profiles were 4.35–38.21% and 4.74–15.71%, respectively, which significantly increased compared to the background profile and other treatment profiles. The oxidizable Cr in the CCA treatment profile decreased with increasing soil depth.

**Figure 8:**
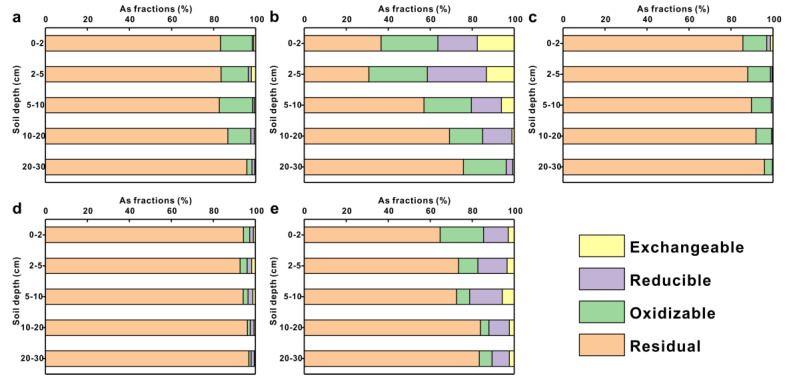
Percentage of exchangeable, reducible, oxidizable, and residual fractions of total As in soil profiles under different preservative–treated planks: (**a**) Background profile; (**b**) CCA-5; (**c**) ACQ-3; (**d**) CA-6; (**e**) CCA plus CA-3.

The residual fraction was the main form of As within the background profile, accounting for 82.70–95.86% of the total content. The oxidizable fraction followed with 2.42–15.81%, while the reducible and exchangeable fractions accounted for a small percentage (Figure 8a). The speciation distributions of As in the profiles under the ACQ and CA treatments were similar to those in the background (Figure 8c,d). In contrast, the percentage of non-residual fractions in the profiles under the CCA and CCA plus CA treatments increased significantly (Figure 8a,e). In the soil profile under CCA treatment, 36.63–87.22%, 11.01–27.84%, 1.55–28.11%, and 0.22–17.59% of residual, oxidizable, reducible, and exchangeable fractions were observed, respectively. Detailed information about As content of each fraction in the soil profiles was presented in the Supplementary Materials (Table S7). Overall, the proportion of residual fractions increased with increasing soil depth for all profiles, and the other fractions decreased with increasing depth.

**Figure 9:**
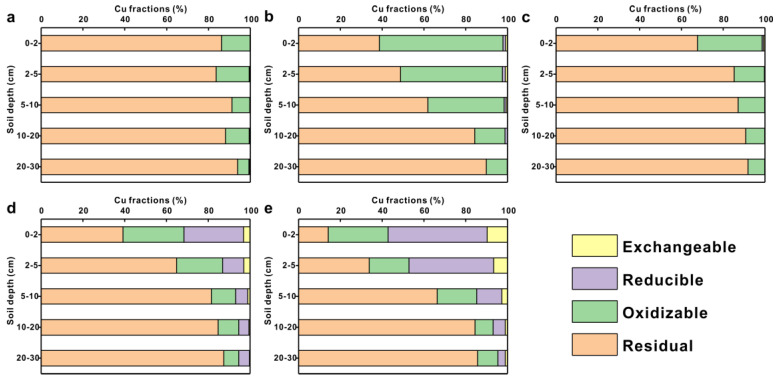
Percentage of exchangeable, reducible, oxidizable, and residual fractions of total Cu in soil profiles under different preservative–treated planks: (**a**) Background profile; (**b**) CCA-5; (**c**) ACQ-3; (**d**) CA-6; (**e**) CCA plus CA-3.

In the background profiles, the Cu speciation in soil horizons was predominantly in the residual and oxidizable fractions, accounting for 83.64–93.88% and 5.46–15.82% of the total content, respectively, while the proportions of the reducible and exchangeable fractions were deficient: less than 1% (Figure 9a). The non-residual fractions in four soil profiles under the preservative treatments decreased with increasing soil depth, and the distribution of each fraction in the profiles was variable: the fraction of Cu in soil profiles under the CCA and ACQ treatment profiles was present in the residue and oxidizable states (38.67–91.54%, 8.37–59.14% (CCA-5); 67.66–91.91%, 7.94–31.03% (ACQ-3) of the total content, respectively). The distribution of reducible and exchangeable states in the CA-6 and CCA plus CA-3 profiles increased significantly (Figure 9b–e). More details about Cu content of each fraction in the soil profiles was presented in the Supplementary Materials (Table S8).

In general, Cr, As, and Cu in soils mainly appeared as residual fractions in all profiles and increased with depth. The proportion of non-residual heavy metals increased in soils after the preservative-treated trestles had been established and decreased with increasing soil depth in the profiles. Meanwhile, the ratio of residual and non-residual fractions decreased with the in-service time of trestles.

## 4. Discussion

### 4.1. Factors Affecting the Distribution of Cr, As, and Cu in Soils

Although the metal(loid)s content of the preservative-treated boardwalks differed in retention in diverse contexts, it was still observed that the treatment with the highest initial concentration had the highest retention, the preservative wood that had been used for a long time retained less metal than those with short-term use from the same batch, and the boardwalks that had been rained on contained less metal(loid) than those that had not been rained on. This result indicated that the loss of metal in the plank road was related to the initial treatment, the in-service time, and the use scenario [45]. In addition, the distribution of metal(loid)s in the soil along the edge of the preserved treatment boardwalk might be influenced by a combination of many factors.

#### 4.1.1. Preservatives Used for Wood Treatment

Elevated levels of Cr, As, and Cu were observed in soils under different boardwalks compared to background concentrations. The differences in metal(loid) contents in the surface soils were mainly attributed to the differences in preservative types in boardwalks. The levels of Cr and As in soils were significantly higher under planks with both CCA- and CCA plus CA-treatments than ACQ- and CA-treatments because ACQ- and CA-treated woods contained only trace amounts of Cr and As. Among the three metal(loid)s, As concentrations were the highest in soils under CCA-treated planks, indicating that As is readily leached from CCA-treated wood [41,68,69] and was retained in the soils. The highest values of Cu content were found in soils under the CCA plus CA treatments, likely as a result of the combined effect of the CCA and CA treatments. The Cu content in soils under the ACQ-treated boards was higher than that under the CCA-treated boards. This result is consistent with a previous study showing that Cu leaching rates were higher in ACQ preservative-treated wood than in CCA-treated wood [31]. In a study to evaluate the loss of different types of preserved wood under field conditions, the leaching rate of Cu was higher in ACQ-treated wood than in CCA-treated wood with different Cu retention levels [32]. The leaching of Cu was no lower in the CA boardwalks than in the other treatments, despite the shorter in-service time, indicating greater leaching of CA preservative-treated wood [34].

Compared to the background profiles, there was a concentration gradient of Cr and As only within the CCA and CCA plus CA profiles, and some of these heavy metals were present in the non-residual fraction. In contrast, the contents of Cr and As within the soil profiles under the ACQ and CA treatments were very low, mainly appearing in the residual fraction, and there was no significant trend with increasing soil depth. The differences in the types of heavy metals exhibited between the profiles were likewise attributed to the different preservative treatments on boardwalks.

#### 4.1.2. Soil Properties

In the SS2 samples, SOM content was positively correlated with the contents of the three elements and reached a significant correlation with Cr and Cu contents (Table 4). It was found that organic matter content was significantly and positively correlated with As and Cr contents, respectively, which was attributed to the fact that organic matter in soils tends to form complexes with heavy metal ions, thus reducing their activity and leading to an increase in soil heavy metal content [66]. Cr, As, and Cu measured in this study exhibited extremely high retention in the surface soils. In contrast, samples collected in areas with sandy soil (average sand content of 95%) contaminated with CCA-treated wood for 5–10 years showed low retention [42]. Usually, Cu is present in a less mobile and biologically effective form in contaminated soils [70]. Only 1–20% of Cu in the soils is bioavailable; however, most Cu is bound to organic matter [71]. Speciation classification studies on Cu in acidic vineyard soils have shown that more than 50% of Cu is organically bound [72]. Therefore, the significant positive correlation between Cr and Cu content and SOM content in the SS2 samples should be due to the formation of complexes between SOM and Cr and Cu, thus reducing bio-effectiveness and mobility and leading to an increase in soil metal content. In the samples of soils at a depth within 0–10 cm, the correlations between metal(loid)s and SOM and soil pH values were not very strong, possibly due to the predominant effect of the extremely high concentration of As, Cr, and Cu in the preservative woods on the corresponding contents in soils.

#### 4.1.3. Geochemical Properties of Cr, As, and Cu

By observing the vertical distribution of Cr, As, and Cu in the profile under the CCA-treated stack, it was found that As was able to migrate significantly and was enriched at soil depths of 0–10 cm, while Cr and Cu were enriched only at 0–5 cm. Furthermore, the speciation distribution of As in the CCA-treated profiles dramatically differed from that of Cr and Cu. In this study, As was less susceptible to transform from an unstable to a stable fraction than Cr and Cu and was the most longitudinally mobile among the three elements. This finding corresponds with those of previous studies, which revealed that when Cr, As, and Cu entered the environment from CCA-treated wood, especially into the soil environment, As was more mobile than Cr and Cu [10,33,73,74].

CCA wood leaching experiments showed that As in leachate was favorable in the form of H_2_AsO_4_^−^ and HAsO_4_^2−^. However, when As entered the soil, As in the leachate was present as As(III) [69]. Adsorption and oxidation reactions of As(III), which is more soluble and mobile than As(V) in soils, are two crucial factors affecting the fate and transport of As in the environment [75]. First, the addition of organic matter may enhance the release of As and increase the migration rate [75,76,77]. Second, As remains soluble in reducing environments, unlike other heavy elements. Anaerobic conditions in soils, as well as increasing pH and decreasing Eh, can promote both the release and migration of As [78,79]. The greater mobility of As in this study might be due to the high organic mass in soils. Once the organic matter increasingly decomposes and the Eh value might decrease, the quantity of As adsorbed initially on the oxide surface is desorbed, and its mobility is enhanced.

In this study, the residual fraction of Cr dominated all contaminated soil profiles, which was consistent with the results of a previous study [80]. After the entrance of exogenous Cr into the soils, the water-soluble and exchangeable fractions of Cr recovered to the control level after six weeks [81]. In this study, the vertical mobility of Cr was low, in accordance with previous studies that have also shown that Cr is the most stable element in preservative wood [82,83,84,85]. Most of the Cr(VI) was reduced to Cr(III) in aged wood [69]. In nature, Cr often exists as Cr(III) (Cr^3+^, CrO_2_^−^) and Cr(VI) (Cr_2_O_7_^2−^ and CrO_4_^2−^) [86]. In soils, Cr(VI) is difficult for soil colloids to absorb, so it has high activity, while Cr(III) is easily adsorbed by soil colloids, so its activity is low [87]. Usually, Cr(VI) is readily converted to Cr(III) via biological and chemical reactions in the natural environment. In addition, natural substances such as soil organic matter, Fe(II), microorganisms, and decomposition products of peroxide compounds such as aldehydes, may reduce Cr(VI) [88]. Therefore, the higher the soil organic matter content is, the higher the capacity and rate of Cr(VI) reduction [89]. Here, Cr may likely be leached out from the CCA-treated wood as Cr(III). Furthermore, the surviving Cr(VI) would also be quickly converted to Cr(III) after entering the soil, so that Cr in the profile mainly exists as less reactive Cr(III) with a low migration rate.

In the profiles under CCA- and ACQ-treated woods, Cu existed mainly in residual and oxidizable fractions. Cu in preservative-treated wood took the form of Cu(II) regardless of the in-service time [90], and Cu mobility and bio-effectiveness in soils are largely controlled by the sorption–desorption behavior of organic and inorganic colloids [91]. The low rate of Cu(II) desorption in soils with high organic matter content is due to the ability of organic matter to form complexes with Cu, which enhances the stability of Cu in soils [67,92,93,94].

#### 4.1.4. In-Service Time

After a long period of physicochemical action, Cu migrated approximately 0.5 m and 5 cm horizontally and vertically in CCA-contaminated soil. However, Cu was enriched only at 0 m horizontally and 2 cm vertically under the shorter use times of the ACQ and CA treatments. The migration distance of Cu was related to the usage time of the boardwalks.

The proportion of reducible and exchangeable Cu in soils under CA- and CCA plus CA-treated planks was significantly higher than that under CCA- and ACQ-treated planks, which was also related to the in-service time of the trestles, i.e., the aging time of heavy metal(loid)s.

The initial sorption reaction on heavy metal(loid)s was rapid after entering the soils, usually within minutes to hours, and was often followed by a long-term response with a slow decrease in leachability, exchangeability, bioefficiency, and toxicity. The whole process is called aging [95,96,97,98]. The exogenous water-soluble fractions of heavy metal(loid)s in soil would result in a decrease in the proportion of metal fractions weakly bound to the soil solid phase (i.e., exchangeable fraction) and an increase in the proportion of other more strongly bound fractions [95,99,100]. In this study, the soil profiles under the CA and CCA plus CA treatments were contaminated by the newly installed CA-treated boardwalks, while the CCA- and ACQ-treated boardwalks were used for much longer than the CA-treated trestles. Cu gradually transformed from a highly mobile to a less-migrated fraction in soils after a long aging period.

#### 4.1.5. Debris Flow

**Figure 10:**
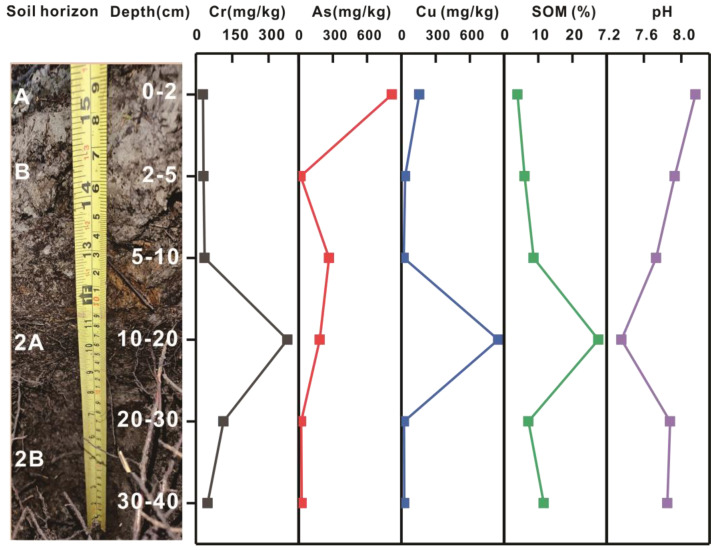
Distribution of Cr, As, Cu, SOM, and pH in the CCA plus CA-2 profile.

In the JNNR, numerous debris-flow gullies have occurred across the Jiuzhaigou Valley [101]. These gullies are frequently impacted by destructive earthquakes, e.g., the Wenchuan earthquake (Ms = 8.0, 2008), Jiuzhaigou earthquake (Ms = 7.0, 2017), and a large number of extra geological hazards, e.g., debris flow gullies and landslides, have broken out the JNNR [102,103,104]. Debris flows may destroy the natural scenery and ecosystems [101], and the original soils under the boards might be washed out or buried by the debris flows in the JNNR. Within the M2 profile, the soil at a depth within 0–10 cm is most likely composed of loose debris and mud brought by debris flows, i.e., the newly formed soil horizons (A and B). The soil at a 10–40 cm depth is brown in color and loamy in texture, of which the soils within the depth between 10 and 20 cm and 20 and 40 cm are the buried surface horizon (2A) and subsurface horizon 2B, respectively. The 2A horizon has more organic matter accumulation and is darker than the 2B horizon while the 2B horizon has less soil organic matter content, a finer texture and more compact structure relative to the 2A horizon (Figure 10). Anomalies in Cr, As, and Cu contents were found in the subsurface soils (10–20 cm) within the M2 profile (Figure 10), which might be because this soil layer was originally a topsoil layer and was interbedded by the debris flow. The new topsoil formed on the debris flow materials would have been affected by the CCA and CA-treated boardwalks concerning As and Cu enrichment in the topsoil at 0–2 cm depth. Meanwhile, it was presumed that the subsoil layer within the depth of 10–20 cm might have been impacted by the CCA-treated board because of the extraordinarily high Cr, As, and Cu contents (Figure 10). This presumption was also evidenced by the highest organic matter content (27.56%) and low pH (7.36) occurring in this layer within the profile (Figure 10), which are typical features in surface soils (see Section 3.2.1).

### 4.2. Environmental Risks and Biological Toxicity of Preservative-Treated Boardwalks

Cr and As contamination was present in the soils beneath the CCA and CCA plus CA treatment boardwalks, and the Cr content was significantly higher than that in the background soils (*p* < 0.05) in the JNNR. However, the environmental risk produced by soil pollution depends not only on the heavy metal(loid) concentration, but also on the metal speciation, ecotoxicity of the metals, aging time, and physical and chemical properties of soil.

The presence of Cr in the soil profiles under the CCA- and CCA plus CA-treated woods in this study was mainly in the residual fraction, and most of the Cr might be in the form of Cr(III), as speculated in the previous paper, both indicating the limited mobility and bio-effectiveness of Cr in the soils. Since Cr(III) is not well absorbed in any pathway, the toxicity of Cr is mainly attributed to Cr(VI) [105]. Cr(VI) is a toxic industrial pollutant classified as a human carcinogen [106]. Human exposure to Cr has been reported to occur through respiratory and dermal contact [107,108]. Inhalation of high Cr(VI) can irritate the nasal mucosa and cause nasal ulcers [109]. The main health issues following the ingestion of Cr(VI) compounds in animals are irritation and ulceration of the stomach and small intestine, anemia, sperm damage, and damage to the male reproductive system. On the other hand, Cr(III) compounds are much less toxic and do not seem to cause these problems [105]. For plants, Cr promotes some plant growth when low doses of Cr are applied [110]. The toxicity of Cr to plants decreases significantly with increasing aging time when high doses are used, and SOM is one of the important factors that promote Cr aging [111]. The prolonged aging time could attenuate the toxicity of Cr on the potential nitrification rate of soil microorganisms [112]. Because Cr originated from CCA-treated boardwalks and the Cr aging time was longer than ten years, coupled with abundant SOM content in the soil in JNNR, it is inferred that the ecological risk of Cr in soils beneath CCA and CCA plus CA-treated boardwalks is low.

As concentrations in surface soils (0–10 cm) under the CCA- and CCA plus CA-treated trestles were ~19.3 and 4.6 times higher than those in background soils, respectively. As-contaminated soils are ecotoxic and carcinogenic [113]. The toxic effects of As are dependent on several factors, of which the chemical speciation (inorganic or organic) and oxidation state are most important. Inorganic As is more toxic than organic As, and As(III) in inorganic is more toxic (2–10 times) than As(V) [114]. Furthermore, As(III) might occur in surface and subsurface environments up to 40% of the total As [115], and leached As(III) from CCA-treated wood was present in soils [69]. In our study, As was more vertically mobile than Cr and Cu, with enrichment depths up to 10 cm, and exchangeable fractions of 0.22–17.59% and 2.25–5.63% were observed in the profiles under the CCA- and CCA plus CA-treated boardwalks, so it is presumed that As was mainly present as As(III). As phytotoxicity is also affected by aging, and the EC50 threshold for As phytotoxicity increased 1.76-fold for soils aged 5 years compared to those aged 0.25 years [97]. Furthermore, EC50 is highly dependent on soil properties [116]. Acidic soils have greater EC values than moderately alkaline soils [97]. In this study, the soils under the CCA and CCA plus CA treatments were mostly moderately alkaline, so the smaller the corresponding EC values were, the greater the toxicity risk. In the JNNR, dermal contact, airborne dusts and contaminated soil might be potential sources of inhalation into the human body. Low-dose and long-term exposure to As can also lead to “arsenic poisoning” in humans [117]. Chronic As poisoning can cause skin damage [118], and inorganic As has the potential to cause skin cancer in humans if ingested over a long period of time [119]. In addition, lung, liver, bladder, and kidney cancers have also been associated with chronic intake of As [120,121,122].

Cu is an essential trace element for plants and humans, but excess Cu plays a negative role in plant growth and human health [123,124,125]. Excess Cu can impair the process of photosynthetic electron transfer in plant cells [126]. Free copper ions in the body’s cells catalyze the production of damaging free radicals [127], causing chest tightness, hemoptysis, nasopharyngeal mucosal congestion, and memory loss in humans. The Cu content of the soils under all CCA plus CA-, ACQ-, CCA-, and CA-treated trestles was 14.7, 10.4, 8.4, and 7.5 times that of background soil, respectively. Similar to other metals, the toxicity and bio-effectiveness of Cu mainly depend on the form of Cu rather than the total amount [128]. Cu in preservative-treated wood is Cu(II), regardless of the duration of use for both conventional and micronized Cu-based preservatives [90]. Similarly, Cu is also present in soils in the form of Cu(II). In our study, the concentration and speciation distribution of Cu in the vertical profiles showed its limited mobility. In addition, the weakly alkaline soil pH and high organic matter in soils of the JNNR were not conducive to Cu migration in the vertical direction [129]. Exchangeable Cu was only present in the profiles under CA- and CCA plus CA-treated trestles (0.25–3.09% and 1.02–9.70% of the total, respectively). After a long period of aging, Cu in the soils under the CCA- and ACQ-treated trestles existed mainly in the residual and organic fractions, and the precipitated and organically bound fractions of Cu were generally nontoxic. Therefore, the ecological risk of Cu in the soils under the CCA and ACQ treatments in this study was low. However, since the newly installed CA-treated boardwalks leach large amounts of Cu, concern should be raised about Cu contamination in the area of new boardwalks.

In summary, the environmental risk of heavy metal(loid)s in soils under CCA treatment was the highest compared to ACQ and CA treatment in this study, largely contributing to the significant toxicity and mobility of As. The risk of Cr and Cu was lower than that of As. For CA treatment, due to the short in-service time, some Cu existed in the soil in a highly effective fraction. The subsequent monitoring of Cu leaching from CA boardwalks and its migration transformation in the soil should be continued.

## 5. Conclusions

In this study, we determined the heavy metal(loid) content in the existing CCA-, ACQ-, and CA-treated trestles in the JNNR and analyzed the total and speciation distribution characteristics in soils. The CCA-treated trestles released large amounts of Cr, As, and Cu into the soils, while the ACQ- and CA-treated trestles released only Cu. Multiple preservative-treated trestles produce great pollution in the underlying surface soil (above 10 cm soil), but this pollution is limited and does not exceed 0.5 m. In general, Cr, As, and Cu in soil were mainly present as residual fractions in all profiles and increased with depth. As is the most mobile element compared to Cr and Cu, and some As is still present in the profile as an exchangeable fraction. In addition, the distribution characteristics and migration behavior of Cr, As, and Cu in the vertical direction are influenced by the in-service time of boardwalks, soil properties (e.g., organic matter content), and geological disasters (e.g., debris flow). Based on the findings of this study, it can be concluded that the types of contaminants were expected to reduce from Cr, As, and Cu to Cu in the future as ACQ and CA-treated boardwalks gradually replaced CCA-treated boardwalks. This resulted in a decrease in total metal content, toxicity, migration, and biological effectiveness, thus reducing the environmental risk. Therefore, early and proper disposal of abandoned and replaced old trestles, especially CCA trestles, is recommended. In the future, ACQ- or CA-treated trestles with less pollutants and fewer ecological risk are recommended instead of CCA-treated trestles. New trestles should be located at the address of the original trestle to the greatest extent possible to avoid expanding the scope of contamination. In addition, long-term Cu contamination monitoring and assessment should be strengthened for new trestles.

## Figures and Tables

**Table 1:** Sampling information.

Sample Name	Sample Types	Quantity	Location
SS2	Surface soil samples (0–2 cm)	35	Zaru Gully, Rize Gully, Shuzheng Gully, Zechawang Gully
	Background (0–2 cm)	7	
SS10	Surface soil samples (0–10 cm)	82	
	Background (0–10 cm)	11	
CCA	Profile	42(8)	Swansea Lake, Panda Center, Lower Season Lake, Primeval Forest
ACQ		15(3)	Primeval Forest, Norilang Parking Lot, Nade, Zaru Monastery, Long Lake, Norilang Waterfall
CA		41(8)	Primeval Forest, Norilang Parking Lot, Nade, Zaru Monastery, Long Lake, Norilang Waterfall
CCA plus CA		17(3)	Upper Seasonal Lake, Middle Seasonal Lake, Shuzheng village
Background		21(4)	Shuzheng village, Zaru Monastery, Primeval Forest, Norilang
W-CCA	Wooden plank roads	8	
W-ACQ		1	
W-CA		5	

CCA plus CA samples referred to the soil contaminated by both the CCA- and CA-treated wooden plank roads.

**Table 2:** Metal(loid) concentrations in the preservative-treated wood in the JNNR and other regions (mg/kg).

Sample Name	Wood Species	Preservative-Treatment	Rained	Wood in-Service Time	Cr	As	Cu	Method	Location	Reference
				Year	mg/kg	mg/kg	mg/kg			
W-CCA-1	Scots pine	CCA	Yes	~19	1228	802	1321	ICP-MS	JNNR, China	This study
W-CCA-2	Scots pine	CCA	Yes	~19	34.3	12.0	229			
W-CCA-3	Scots pine	CCA	Yes	~19	4359	781	2667			
W-CCA-4	Scots pine	CCA	Yes	~19	8077	2339	2667			
W-CCA-5	Scots pine	CCA	Yes	~19	553	247	1382			
W-CCA-6	Scots pine	CCA	No	10	3406	1302	1223			
W-CCA-7	Scots pine	CCA	Yes	10	1677	714	1358			
W-CCA-8	Scots pine	CCA	Yes	~19	2920	1033	1644			
W-CCA-9	Scots pine	CCA	Yes	~8	-	33.6	-	XRF	JNNR, China	[45]
W-CCA-10	Scots pine	CCA	Yes	~8	-	2546	-			
W-CCA-11	Scots pine	CCA	Yes	~8	-	2734	-			
W-CCA-12	Scots pine	CCA	Yes	~8	-	2014	-			
W-CCA-13	Southern Yellow Pine	CCA	Yes	Unknown	1650	455	1100	ICP-MS	Miami, USA	[32]
W-CCA-14	Southern Yellow Pine	CCA	Yes	Unknown	2680	1440	1570			
W-CCA-15	Southern Yellow Pine	CCA	Yes	25	14500	20700	7300			
W-CCA-16	Unknown	CCA	Unknown	Unknown	4944	4309	2806	ICP-ES	South Korea	[56]
W-CCA-17	Scots pine	CCA	Unknown	Unknown	-	-	1060	ICP	Turkey	[57]
W-CCA-18	Red pine	CCA	Unknown	0 (Fresh)	-	-	2616	ICP-AES	Toronto, Canada	[58]
W-ACQ-1	Southern pine	ACQ	Yes	5~10	24.1	11.6	5234	ICP-MS	JNNR, China	This study
W-ACQ-2	Unknow	ACQ	Unknown	Unknown	46	17	16255	ICP-ES	South Korea	[56]
W-ACQ-3	Southern Yellow Pine	ACQ	No	0	-	-	1780	ICP-MS	Miami, USA	[32]
W-ACQ-4	Unknow	ACQ	Unknown	Unknown	-	--	1890	ICP-AES	Canada	[16]
W-ACQ-5	Scots pine	ACQ	Unknown	Unknown	-	-	104	ICP	Turkey	[57]
W-ACQ-6	Spruce pine fir	ACQ	No	0	-	-	1761	ICP-AES	Toronto, Canada	[58]
W-ACQ-7	Southern pine	ACQ	Yes	1	-	-	6433	ICP-AES		
W-CA-1	Southern pine	CA	Yes	0~2	5.5	3.2	4385	ICP-MS	JNNR, China	This study
W-CA-2	Southern pine	CA	No	0	16.0	6.8	8016	ICP-MS		
W-CA-3	Southern pine	CA	Yes	0~2	11.4	2.9	6037	ICP-MS		
W-CA-4	Southern pine	CA	Yes	0~2	6.9	2.9	4032	ICP-MS		
W-CA-5	Southern pine	CA	Yes	0~2	4.0	1.6	4605	ICP-MS		
W-CA-6	Unknown	CA	Unknown	Unknown	-	-	1190	ICP-AES	Canada	[16]
W-CA-7	Red pine	CA	Yes	Unknown	-	-	6125	ICP-AES	Toronto, Canada	[58]
W-CA-8	Spruce-pine fir	CA	No	0	-	-	2099			
W-CA-9	Southern pine	CA	Yes	1	-	-	5436			

“-” is unmeasured.

**Table 4:** Matrix of correlation coefficients between metal(loid)s and soil pH and SOM content in surface soils under CCA-treated boardwalks.

Surface Soil Sample (Depth)	Properties	Cr	As	Cu
SS2 (0–2 cm)	SOM	0.603 *	0.061	0.622 *
	pH	−0.204	0.142	−0.237
SS10 (0–10 cm)	SOM	−0.147	0.488	0.495
	pH	0.231	−0.194	−0.235

* indicates a significant correlation (*p* < 0.05).

## Data Availability

Not applicable.

## Supplementary Materials

The following supporting information can be downloaded at: https://www.mdpi.com/article/10.3390/toxics11030249/s1, Table S1: The SOM (%) in the soil profiles under different preservative-treated planks; Table S2: The pH in the soil profiles under different preservative-treated planks. Table S3: The total Cr content (mg/kg) in the soil profiles under different preservative-treated planks. Table S4: The total As content (mg/kg) in the soil profiles under different preservative-treated planks. Table S5: The total Cu (mg/kg) content in the soil profiles under different preservative-treated planks. Table S6: Cr content (mg/kg) of each fraction in the soil profiles under different preservative-treated plank. Table S7: As content (mg/kg) of each fraction in the soil profiles under different preservative-treated plank. Table S8: Cu content (mg/kg) of each fraction in the soil profiles under different preservative-treated plank.
