# Supplementary material for: Distribution and Speciation of Heavy Metal(loid)s in Soils under Multiple Preservative-Treated Wooden Trestles
Source: Toxics. 2023 Mar 7;11(3):249. doi: 10.3390/toxics11030249 (PMC10056422; doi:10.3390/toxics11030249)
Supplement: Supplementary file 1 [file toxics-11-00249-s001.zip › toxics-2215816-supplementary.pdf]

Table S1 The SOM (%) in the soil profiles under different preservative-treated planks

Treatments / Soil depth	CCA-1	CCA-2	CCA-3	CCA-4	CCA-5	CCA-6	CCA-7	CCA-8	ACQ-1	ACQ-2	ACQ-3	CA-1	CA-2	CA-3	CA-4	CA-5	CA-6	CA-7	CA-8	CCA plus CA-1	CCA plus CA-2	CCA plus CA-3	BG1	BG2	BG3	BG4
0-2 cm	41.92	27.15	15.75	10.91	33.82	15.66	50.22	46.01	39.73	9.00	12.74	10.32	15.65	13.45	3.45	9.80	15.45	10.38	17.34	23.78	3.98	10.34	27.86	22.65	51.20	33.90
2-5 cm	45.90	22.69	14.27	10.46	32.27	26.53	43.53	48.11	35.37	7.87	9.56	11.80	15.44	17.05	3.45	16.06	17.91	11.25	16.52	29.53	6.03	9.71	16.76	21.54	58.27	32.19
5-10 cm	31.64	17.70	12.19	11.58	29.42	46.86	22.67	18.64	9.34	8.44	8.94	11.64	13.22	22.46	2.94	14.80	14.25	17.76	12.27	25.41	8.68	9.65	10.41	19.28	52.80	27.50
10-20 cm	31.37	14.74	14.76	9.56	10.42	38.26	51.81	2.44	3.15	7.51	8.17	17.94	11.78	15.25	2.64	13.33	12.21	10.83	8.04	20.15	27.56	9.66	6.80	16.80	3.88	9.16
20-30 cm	25.39	12.17	12.91	9.27	9.83	38.17	23.64	2.18	3.44	5.37	5.49	16.13	9.91	11.46	2.29	10.18	10.75	8.07	5.61	8.30	7.14	9.13	5.65	11.21	1.93	6.26
30-40 cm					6.26			2.91										6.15		8.31	11.58				1.90	

Table S2 The pH in the soil profiles under different preservative-treated planks

Soil depth \ Treatments	CCA-1	CCA-2	CCA-3	CCA-4	CCA-5	CCA-6	CCA-7	CCA-8	ACQ-1	ACQ-2	ACQ-3	CA-1	CA-2	CA-3	CA-4	CA-5	CA-6	CA-7	CA-8	CCA plus CA-1	CCA plus CA-2	CCA plus CA-3	BG1	BG2	BG3	BG4
0-2 cm	7.16	7.19	7.65	7.78	7.00	7.58	5.47	7.04	6.76	7.83	7.73	7.91	7.72	7.46	8.19	8.02	7.44	7.12	7.44	7.36	8.15	7.55	5.22	6.55	6.40	4.78
2-5 cm	7.16	7.45	7.74	7.92	7.41	7.19	5.46	7.38	6.88	7.94	8.06	7.74	7.20	7.34	8.15	7.53	7.18	7.04	7.56	7.00	7.93	7.73	4.41	6.97	5.08	5.03
5-10 cm	7.54	7.52	7.86	7.90	7.36	6.86	7.26	7.91	7.36	7.90	8.12	7.75	7.60	7.50	8.25	7.67	7.66	7.39	7.70	7.04	7.73	7.84	4.82	7.46	6.50	6.48
10-20 cm	7.20	7.63	7.73	7.82	7.91	6.90	7.00	8.20	8.16	7.87	8.19	7.84	7.84	7.37	8.25	7.78	7.71	6.90	7.91	7.41	7.36	7.80	6.30	7.59	7.95	6.93
20-30 cm	7.35	7.76	7.86	7.93	7.90	7.16	7.12	8.23	8.29	8.03	8.28	7.89	7.89	7.64	8.47	7.91	7.89	6.65	8.05	7.74	7.88	7.86	7.65	7.76	8.23	7.55
30-40 cm					7.99			8.26										6.46		7.92	7.85				8.25	

Table S3 The total Cr content (mg/kg) in the soil profiles under different preservative-treated planks

Treatments / Soil depth	CCA-1	CCA-2	CCA-3	CCA-4	CCA-5	CCA-6	CCA-7	CCA-8	ACQ-1	ACQ-2	ACQ-3	CA-1	CA-2	CA-3	CA-4	CA-5	CA-6	CA-7	CA-8	CCA plus CA-1	CCA plus CA-2	CCA plus CA-3	BG1	BG2	BG3	BG4
0-2 cm	232.31	105.87	121.79	98.09	103.47	51.96	134.76	273.55	35.69	94.24	69.65	74.59	65.10	71.50	66.63	46.38	74.08	75.44	63.42	102.48	29.59	84.62	56.01	67.43	16.56	20.58
2-5 cm	160.96	97.88	98.62	89.13	79.33	65.92	129.10	263.82	30.12	87.03	73.50	72.61	67.50	63.24	67.46	64.07	73.37	73.86	61.05	102.06	31.90	88.76	76.08	72.45	15.68	30.00
5-10 cm	124.22	93.50	98.02	97.78	95.18	61.75	73.95	57.16	32.60	94.64	74.10	65.46	68.64	57.46	65.88	64.64	73.65	71.83	61.28	107.06	36.25	95.80	83.65	74.97	27.42	38.21
10-20 cm	110.55	88.82	132.90	85.58	56.27	49.47	46.94	26.02	55.24	84.69	69.66	69.71	53.37	81.03	42.91	57.59	73.83	79.39	49.50	75.98	377.63	73.96	84.28	79.72	36.17	40.34
20-30 cm	100.13	89.63	121.06	98.97	59.68	40.01	32.91	38.91	31.78	74.29	66.68	65.40	67.32	71.69	63.85	60.23	74.39	76.37	53.63	102.11	114.22	74.24	92.90	73.03	37.85	58.32
30-40 cm					43.07			31.15										78.34		96.41	48.66				35.52	

Table S4 The total As content (mg/kg) in the soil profiles under different preservative-treated planks

Treatments / Soil depth	CCA-1	CCA-2	CCA-3	CCA-4	CCA-5	CCA-6	CCA-7	CCA-8	ACQ-1	ACQ-2	ACQ-3	CA-1	CA-2	CA-3	CA-4	CA-5	CA-6	CA-7	CA-8	CCA plus CA-1	CCA plus CA-2	CCA plus CA-3	BG1	BG2	BG3	BG4
0-2 cm	1036.32	48.01	104.68	775.79	213.20	24.17	107.84	243.87	44.82	182.71	19.33	28.72	31.41	28.32	18.56	22.92	29.79	28.02	24.50	61.74	817.35	48.39	26.83	24.09	5.51	6.52
2-5 cm	775.61	48.52	73.03	711.05	163.20	59.62	151.81	191.11	33.39	306.31	18.18	29.65	33.22	29.90	18.24	26.16	29.22	24.38	23.87	76.61	20.00	55.00	28.24	23.89	5.24	10.11
5-10 cm	821.48	327.02	41.74	471.57	106.17	101.40	136.61	115.11	34.31	466.35	16.96	29.55	32.71	27.78	19.27	25.79	27.81	28.26	21.60	37.93	266.96	89.00	34.77	25.59	6.97	12.42
10-20 cm	168.19	34.18	195.31	153.06	48.32	25.70	14.24	14.11	26.30	259.01	18.07	28.63	26.38	32.66	17.82	24.39	28.09	41.23	19.34	22.78	186.40	46.13	35.06	24.21	7.79	12.69
20-30 cm	134.20	29.16		114.45	30.24	21.62	16.51	130.08	14.34	63.29	17.66	27.71	20.63	30.04	14.45	24.46	29.42	25.02	17.62	17.76	23.81	45.35	35.48	25.89	11.40	19.00
30-40 cm					21.25			178.59				--						26.34		17.02	28.07				8.76	

Table S5 The total Cu (mg/kg) content in the soil profiles under different preservative-treated planks

Treatments / Soil depth	CCA-1	CCA-2	CCA-3	CCA-4	CCA-5	CCA-6	CCA-7	CCA-8	ACQ-1	ACQ-2	ACQ-3	CA-1	CA-2	CA-3	CA-4	CA-5	CA-6	CA-7	CA-8	CCA plus CA-1	CCA plus CA-2	CCA plus CA-3	BG1	BG2	BG3	BG4
0-2 cm	552.02	43.89	59.91	53.41	107.74	21.17	212.92	311.20	35.89	657.41	96.35	270.66	270.66	382.45	62.16	328.33	379.55	27.47	100.97	938.14	158.71	1199.39	23.23	18.03	9.00	12.70
2-5 cm	634.56	39.28	39.47	49.43	66.08	69.77	258.97	264.48	24.37	401.31	26.88	89.58	89.58	91.56	27.13	172.87	97.88	19.41	39.21	1329.01	33.05	803.56	20.87	23.27	8.62	13.02
5-10 cm	48.40	38.08	28.93	36.04	70.74	114.02	33.20	26.49	7.51	384.16	22.83	83.58	83.58	66.04	24.39	46.83	45.39	25.21	21.08	92.25	21.27	176.20	23.89	20.07	10.23	14.30
10-20 cm	23.83	30.02	85.92	52.93	23.09	20.11	12.18	5.40	3.95	249.15	22.34	63.64	63.64	125.14	20.97	30.35	35.35	17.92	18.23	23.42	848.37	61.86	22.62	19.46	18.76	21.12
20-30 cm	22.96	23.52	55.23	35.25	19.58	24.43	9.54	27.34	5.40	166.65	17.99	40.77	40.77	72.89	12.77	21.78	30.91	16.82	17.52	23.52	24.02	56.90	21.71	19.05	5.20	18.21
30-40 cm					16.14			9.16										16.73		39.40	27.32				4.95	

Table S6 Cr content (mg/kg) of each fraction in the soil profiles under different preservative-treated plank

Treatments	Soil depth (cm)	Residual	Oxidizable	Reducible	Exchangeable
CCA-5	0-2	60.16	39.62	3.16	0.76
	2-5	68.86	20.40	2.25	0.65
	5-10	80.08	21.34	1.66	0.22
	10-20	60.61	4.88	0.96	0.05
	20-30	63.09	3.38	0.27	0.04
	30-40	58.78	2.68	0.16	0.04
ACQ-3	0-2	77.43	4.26	0.43	0.12
	2-5	78.88	3.93	0.31	0.08
	5-10	83.99	4.22	0.28	0.09
	10-20	80.02	3.22	0.24	0.07
	20-30	73.70	2.73	0.19	0.13
CA-6	0-2	76.07	4.08	1.54	0.09
	2-5	77.18	4.28	1.41	0.11
	5-10	75.91	4.42	1.68	0.10
	10-20	76.41	4.47	2.08	0.08
	20-30	70.13	3.97	2.20	0.06
CCA plus CA-3	0-2	75.80	9.80	3.53	0.44
	2-5	87.37	12.46	4.49	0.49
	5-10	80.12	15.78	3.97	0.55
	10-20	75.05	3.81	1.41	0.13
	20-30	70.57	4.32	1.58	0.19
Background	0-2	60.82	3.65	0.22	0.71
	2-5	74.04	2.86	0.21	0.18
	5-10	80.76	2.41	0.37	0.01
	10-20	83.46	2.67	0.70	0.02
	20-30	76.13	1.96	0.24	0.01

Table S7 As content (mg/kg) of each fraction in the soil profiles under different preservative-treated plank

Treatments	Soil depth (cm)	Residual	Oxidizable	Reducible	Exchangeable
CCA-5	0-2	82.14	60.57	42.10	39.45
	2-5	47.71	43.13	43.55	20.54
	5-10	72.06	28.39	18.13	7.66
	10-20	36.33	8.36	7.25	0.60
	20-30	25.80	6.91	1.09	0.22
	30-40	21.52	2.72	0.38	0.05
ACQ-3	0-2	17.74	2.35	0.36	0.25
	2-5	15.16	1.87	0.16	0.06
	5-10	15.86	1.66	0.11	0.03
	10-20	18.66	1.52	0.12	0.02
	20-30	18.98	0.73	0.05	0.01
CA-6	0-2	29.94	0.91	0.59	0.29
	2-5	29.96	1.10	0.73	0.55
	5-10	25.23	0.63	0.62	0.32
	10-20	26.11	0.37	0.45	0.20
	20-30	27.60	0.30	0.45	0.14
CCA plus CA-3	0-2	31.26	9.95	5.71	1.35
	2-5	40.95	5.17	7.72	1.92
	5-10	67.66	5.81	14.52	5.25
	10-20	43.12	2.10	5.04	1.16
	20-30	37.37	2.75	3.67	1.05
Background	0-2	20.01	3.61	0.18	0.22
	2-5	21.72	4.15	0.22	0.17
	5-10	28.12	3.51	0.52	0.24
	10-20	27.31	0.69	0.39	0.10
	20-30	24.41	3.76	0.44	0.60

Table S8 Cu content (mg/kg) of each fraction in the soil profiles under different preservative-treated plank

Treatments	Soil depth (cm)	Residual	Oxidizable	Reducible	Exchangeable
CCA-5	0-2	36.41	55.69	1.18	0.88
	2-5	28.57	28.64	0.78	0.61
	5-10	47.37	28.02	0.72	0.49
	10-20	19.51	3.33	0.28	0.00
	20-30	19.87	2.21	0.04	0.00
	30-40	16.71	1.53	0.02	0.00
ACQ-3	0-2	75.10	34.44	0.74	0.71
	2-5	24.02	4.07	0.08	0.00
	5-10	20.15	2.91	0.04	0.00
	10-20	22.52	2.24	0.04	0.00
	20-30	18.94	1.64	0.03	0.00
CA-6	0-2	124.19	92.71	90.70	9.80
	2-5	62.79	21.39	9.82	2.88
	5-10	44.17	6.34	3.09	0.61
	10-20	33.66	3.87	1.96	0.20
	20-30	30.02	2.46	1.75	0.09
CCA plus CA-3	0-2	148.38	300.22	497.89	101.69
	2-5	259.26	146.60	312.28	50.42
	5-10	134.12	38.09	24.38	5.36
	10-20	58.26	5.95	4.00	0.74
	20-30	54.41	6.18	2.25	0.65
Background	0-2	20.43	3.23	0.00	0.03
	2-5	22.32	2.07	0.03	0.03
	5-10	23.93	3.08	0.05	0.09
	10-20	23.27	1.35	0.10	0.06
	20-30	20.29	3.84	0.02	0.11
